# A case of minimum invasive debranch thoracic endovascular aortic repair for isolated left vertebral artery: complete revascularization without artificial vessels via a single small incision

**DOI:** 10.1093/jscr/rjae595

**Published:** 2024-10-05

**Authors:** Masahiro Tsutsui, Kazuki Miyatani, Kentaro Shirakura, Yuki Setogawa, Fumitaka Suzuki, Hiroyuki Miyamoto, Ryo Okubo, Ryohei Ushioda, Shingo Kunioka, Natsuya Ishikawa, Norihumi Otani, Hiroyuki Kamiya

**Affiliations:** Department of Cardiac Surgery, Asahikawa Medical University, Asahikawa 078-8510, Hokkaido, Japan; Department of Cardiac Surgery, Asahikawa Medical University, Asahikawa 078-8510, Hokkaido, Japan; Department of Cardiac Surgery, Asahikawa Medical University, Asahikawa 078-8510, Hokkaido, Japan; Department of Cardiac Surgery, Asahikawa Medical University, Asahikawa 078-8510, Hokkaido, Japan; Department of Cardiac Surgery, Asahikawa Medical University, Asahikawa 078-8510, Hokkaido, Japan; Department of Cardiac Surgery, Asahikawa Medical University, Asahikawa 078-8510, Hokkaido, Japan; Department of Cardiac Surgery, Asahikawa Medical University, Asahikawa 078-8510, Hokkaido, Japan; Department of Cardiac Surgery, Asahikawa Medical University, Asahikawa 078-8510, Hokkaido, Japan; Department of Cardiac Surgery, Asahikawa Medical University, Asahikawa 078-8510, Hokkaido, Japan; Department of Cardiac Surgery, Asahikawa Medical University, Asahikawa 078-8510, Hokkaido, Japan; Department of Cardiovascular Surgery, Sapporo Higashi Tokushukai Hospital, Sapporo 065-0033 Hokkaido, Japan; Department of Cardiac Surgery, Asahikawa Medical University, Asahikawa 078-8510, Hokkaido, Japan

**Keywords:** isolated left vertebral artery, thoracic endovascular aortic repair, Willis arterial circle

## Abstract

Isolated left vertebral artery (ILVA) is one of the most frequent vertebral abnormalities. When performing thoracic endovascular aortic repair (TEVAR), the ILVA may have to be closed depending on the position of the stent graft; in these cases, the decision to reconstruct the ILVA depends on the state of cerebral blood flow. Here, we report a case of a 68-year-old male, in whom the Willis arterial circle was incomplete; we therefore performed a reconstructive method during zone 2-landing TEVAR that ensured ILVA and left subclavian artery blood flow without the use of artificial vessels. Only one supraclavicular incision was required for reconstruction. This method has some procedural difficulties; however, it does not use artificial blood vessels and can be performed with a single incision.

## Introduction

Isolated left vertebral artery (ILVA) is one of the most frequent abnormalities of vertebral origin. When performing thoracic endovascular aortic repair (TEVAR) with zone 2 landing requires occlusion of the ILVA origin, a wound is often placed in the left neck and left subclavian region and revascularization is performed by anastomosing the left vertebral artery (LVA) to artificial vessels bypassed from the left common carotid artery (LCCA) to the left subclavian artery (LSA); anastomosis of the LVA directly to the LCCA can also be performed. However, these methods result in two incisions and may make dissection of the LVA difficult in patients where the ILVA enters at a low position below the transverse process of the vertebral body. We report a case of zone 2-landing TEVAR in a patient with ILVA that allowed revascularization of a single supraclavicular incision without the use of artificial vessels. This method appears to be one of the most effective for revascularization in patients with specific anatomical features.

## Case report

**Figure 1:**
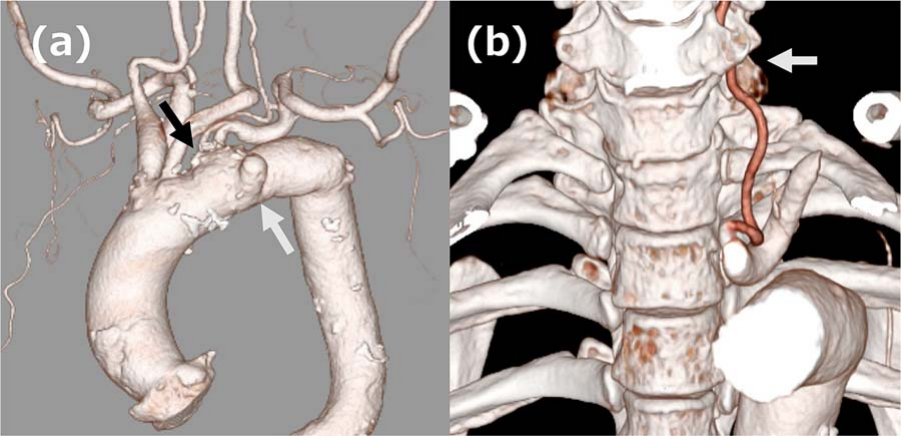
(a) Three-dimensional computed tomography (3DCT) image. The white arrow indicates the aortic aneurysm, and the black arrow indicates ILVA. (b) 3DCT image. The white arrow indicates the entry of ILVA under the transverse process of the vertebra at the sixth cervical vertebra.

**Figure 2:**
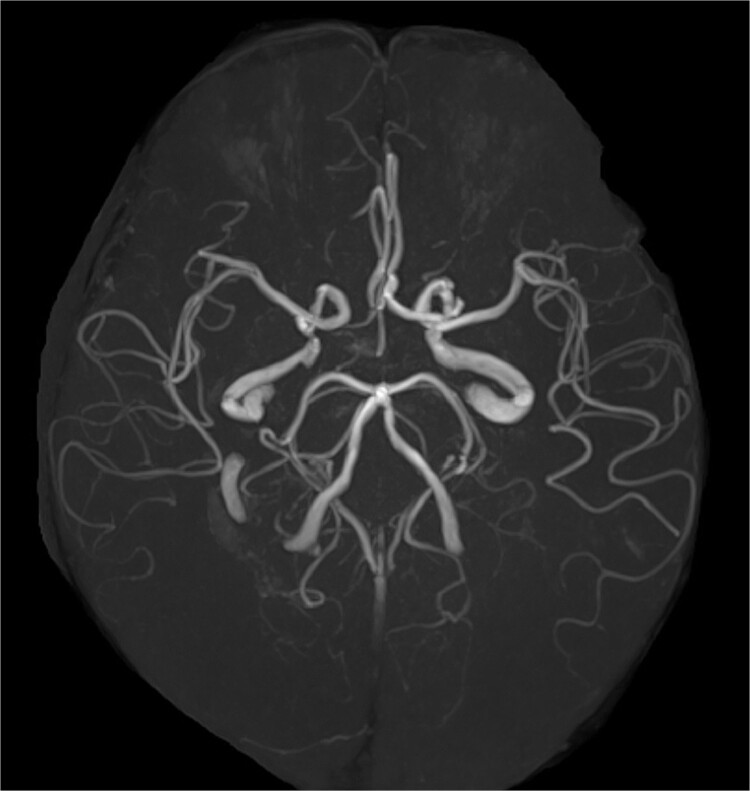
Magnetic resonance angiography image. The Willis arterial circle is incomplete.

A 68-year-old male patient had undergone distal aortic arch replacement with lateral thoracotomy 13 years earlier for chronic distal aortic arch dilatation of a type B aortic dissection of unknown onset. Three years ago, a saccular aortic aneurysm, probably arising from the anastomosis site, was observed; as it had a tendency to dilate over time, surgical intervention to prevent rupture was decided upon. Although the patient was scheduled for TEVAR, his anatomy showed an ILVA with its origin in a position of forced occlusion due to the stent graft landing in zone 2 (Fig. 1a). In most cases, the entry of the ILVA under the transverse process of the vertebra is at the fifth cervical vertebra; however, in this case, the entry was from the sixth cervical vertebra (Fig. 1b). Magnetic resonance angiography showed poor delineation of the bilateral posterior communicating arteries, and the Willis arterial circle was likely nonfunctional, suggesting that revascularization of the ILVA would be beneficial for neurological prognosis (Fig. 2).

**Figure 3:**
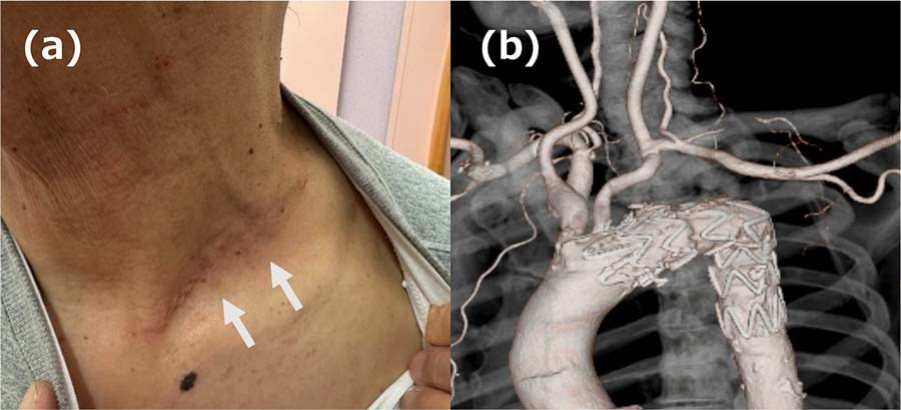
(a) Picture of the wound. The white arrow indicates the wound. (b) 3DCT image. All reconstruction was performed without artificial vessels.

The left supraclavicular approach was chosen because the incision on the left side of the neck was considered difficult to mobilize with ILVA; this approach is typically used to bypass the LSA from the LCCA by using an artificial vessel. In this case, we decided to mobilize the LSA during the mobilization of the ILVA and perform revascularization by anastomosing the LSA directly to the LCCA. A 7 cm incision was made over the left clavicle, and the LCCA, LSA, and ILVA were mobilized. The central side of the LSA was ligated and cut, and it was anastomosed to the LCCA; the ILVA was anastomosed to the LSA at a short distance from the LSA/LCCA anastomosis. TEVAR was placed in zone 2 using Relay®pro (Terumo, Tokyo, Japan) and no endoleak was identified on intraoperative contrast. The patient had an uneventful postoperative course; the wound healed discreetly and cleanly (Fig. 3a), and a postoperative computed tomography scan confirmed that the revascularization was functioning without endoleak (Fig. 3b). The patient was discharged on postoperative Day 8.

## Discussion

In our case, TEVAR was performed after reconstruction of the LSA and ILVA. With regard to reconstruction of the LSA, there seems to be no major objection to reconstruction, for example because simple closure increases the risk of spinal cord ischemia [1]. However, there is no consensus on reconstruction of the ILVA when performing TEVAR. In recent years, there have been some reports on ILVA reconstruction, and one factor that may determine its need is cerebral blood flow. Ding *et al.* reported that in TEVAR for type B aortic dissection with ILVA, reconstruction was performed in patients with dominant ILVA or qual right vertebral artery and ILVA but without preservation of the Willis arterial circle [2]. Van der Weijde *et al.* also reported the management of aortic aneurysm in patients with a distal arch aortic aneurysm with an incomplete Willis arterial circle due to aversion to ILVA occlusion [3]. In our case, the Willis arterial circle was not preserved, and reconstruction was performed to improve the neurological prognosis.

The advantages of the ILVA reconstruction method used in this case are that it is easy to secure the vertebral artery entering the subtransverse process from a low position, it can be completed with a single incision, and it does not require an artificial vessel; also, the procedure follows a cutaneous dividing line and has good cosmetic qualities. Yang *et al.* reported on the safety and favorable results of LCCA-LSA bypass and ILVA reconstruction using a supraclavicular incisional approach [4]. Their superior method used artificial vessels for bypass; our method used direct anastomosis of the LSA to the LCCA without artificial vessels, which inevitably required adequate vessel mobilization. Mobilization of the LSA is inevitably difficult due to the deep field of view and narrow surgical area. Therefore, a thorough understanding of the surrounding anatomy is necessary. Particular care must be taken not to damage the transverse nerves running in front of the anterior scalene muscle. Care must also be taken not to damage the vagus nerve when mobilizing the LCCA, although the visual field is good.

With regard to the advantage of not using artificial vessels, it is possible to extend the indication to ILVAs entering the transverse process from a higher position. The indications for reconstruction of the vertebral artery by this method can be extended to cases with normal anatomy where artificial vessels close to the body surface should be avoided, although the time and effort required for reconstruction is increased.

## Conclusion

Non-prosthetic revascularization technique via a small single incision was useful for ILVA patient with debranch TEVAR. Although the indications for this procedure should be carefully evaluated owing to its difficulty, it is thought to be highly beneficial to patient outcomes if stable results are achieved.
